# Association of inflammation and endothelial dysfunction with metabolic syndrome, prediabetes and diabetes in adults from Inner Mongolia, China

**DOI:** 10.1186/1472-6823-11-16

**Published:** 2011-10-11

**Authors:** Angela M Thompson, Yonghong Zhang, Weijun Tong, Tan Xu, Jing Chen, Li Zhao, Tanika N Kelly, Chung-Shiuan Chen, Lydia A Bazzano, Jiang He

**Affiliations:** 1 Department of Epidemiology, Tulane University School of Public Health and Tropical Medicine, New Orleans, Louisiana, USA; 2 Department of Epidemiology, School of Radiation Medicine and Public Health, Medical College of Soochow University, Suzhou, Jiangsu, PR China; 3 Department of Medicine, Tulane University School of Medicine, New Orleans, Louisiana, USA; 4 Tongliao Center for Disease Prevention and Control, Tongliao, Inner Mongolia, PR China

**Keywords:** metabolic syndrome, diabetes, inflammation, endothelial dysfunction, C-reactive protein, intercellular adhesion molecule-1, E-selectin

## Abstract

**Background:**

We examined the association of biomarkers of inflammation and endothelial dysfunction with diabetes and metabolic syndrome (MetS) in persons from Inner Mongolia.

**Methods:**

A cross-sectional study was conducted among 2,536 people aged 20 years and older from Inner Mongolia, China. Overnight fasting blood samples were obtained to measure plasma concentrations of high sensitivity C-reactive protein (hsCRP), soluble inter-cellular adhesion molecule-1 (sICAM-1), sE-selectin, angiotensin II, high density lipoprotein cholesterol, triglycerides, and blood glucose. Waist circumference and blood pressure were measured by trained staff. MetS was defined according to the modified ATP III definition for Asians. Elevated level of the biomarker was defined as values in the upper tertile of the distribution. Participants were categorized into one of four groups based on the presence or absence of metabolic and glycemic abnormalities: 1) free of prediabetes, diabetes and MetS (reference group), 2) prediabetes or diabetes only, 3) MetS without prediabetes or diabetes, and 4) MetS plus prediabetes or diabetes. The multivariable models are adjusted for age, gender, smoking, drinking, family history of hypertension, and body mass index.

**Results:**

Among study participants, 18.5% had prediabetes, 3.6% had diabetes, and 27.4% of the entire study population had 3 or more components of the MetS. Elevated hsCRP was associated with an increased odds of prediabetes or diabetes only, MetS without prediabetes or diabetes, and MetS plus prediabetes or diabetes with multivariable adjusted odds ratios (95% confidence intervals) of 2.3 (1.7-3.1), 3.0 (2.4-3.8), and 5.8 (4.5-7.5), respectively. Elevated sICAM-1 was associated with increased odds (95% CI) of prediabetes or diabetes only (2.1, 1.6-2.9) and MetS plus prediabetes or diabetes (4.2, 3.2-5.3) but was not associated with MetS alone. Elevated sE-selectin was associated with a modestly increased risk of MetS (OR 1.7, 95% CI 1.4-2.2). Elevated levels of Angiotensin II were not associated with the MetS plus prediabetes or diabetes in this study.

**Conclusions:**

Diabetes and the MetS are common in the Inner Mongolia population. The biomarkers of inflammation and endothelial dysfunction are associated with increased risk for diabetes and MetS in this population. These results are consistent with results from other populations.

## Background

The metabolic syndrome (MetS) is common in the United States and has become more prevalent worldwide in recent years. While much has been written about the MetS in US and European populations, less is known about the epidemiology of MetS in non-western countries [1-3]. In a nationally representative sample of adults in China, the age-standardized prevalence of MetS was reported to be higher in women (29.1%) than men (17.7%), in northern (30.9%) compared to southern (18.2%) China, and in urban areas (31.0%) compared to rural (21.6%) areas [4]. It is estimated that 66% of persons with diabetes also have the MetS [5]. In Chinese adults, the age-standardized prevalence of prediabetes, diabetes and MetS were reported to be 15.5%, 9.7%, and 23.3%, respectively [4,6].

MetS is characterized by the presence of multiple cardiovascular risk factors including abdominal obesity, dyslipidemia, elevated glucose and elevated blood pressure [7]. The risk of cardiovascular disease (CVD) morbidity and mortality is elevated among those with MetS [8-10], and as the number of metabolic abnormalities increase, the risks of stroke, [11], chronic kidney disease[12] and CVD [13,14] increase. However, not all of the components of MetS confer the same amount of risk. In Western populations, elevated blood glucose may be the primary force driving the development of MetS and subsequent CVD risk [8]. Diabetes confers a high risk for CVD mortality that is modified little by the addition of the other MetS risk factors [15].

Biomarkers of inflammation and endothelial dysfunction have been associated with diabetes [16-19] and the MetS [20-22]. They may explain as much as 43% of the excess risk for CVD mortality among persons with diabetes [16] and up to 26% of the association between MetS and coronary artery disease [23]. However, the associations reported have been inconsistent in Western and Asian populations [16,20,24-30]. To our knowledge, no previous study has examined this association in persons of Mongolian ethnicity. In the current study, we examined the association between biomarkers of inflammation and endothelial dysfunction with categories of metabolic or glycemic abnormality in a lean, farming population from Inner Mongolia, China.

## Methods

### Subjects

A cross-sectional study was conducted between 2002 and 2003 in Inner Mongolia, an autonomous region in north China. The methods for selection of study participants and data collection have been described elsewhere [31]. Briefly, individuals aged 20 years and older were recruited from 32 villages in two adjacent townships located in Kezuohou Banner and Naiman Banner in Inner Mongolia. The majority of local residents were Mongolians who had lived there for many generations, their professions were farmers and herdsmen and they maintained a traditional diet that was high in fat and salt. Out of 3,475 eligible people, a total of 2,589 individuals chose to participate. Those who did not have a measurement for one or more of the components for MetS were excluded (n = 53) and a total of 2,536 persons were included in this analysis. Written informed consent was obtained for all study participants. This study was approved by the ethics committee at Soochow University in China and institutional review board at Tulane University Health Sciences Center in the United States.

### Data Collection

Trained staff interviewed participants in Chinese using a standard questionnaire to obtain information on demographic characteristics, medical history, and lifestyle risk factors such as cigarette smoking and alcohol consumption. Three sitting blood pressure measurements were taken for each participant using a mercury sphygmomanometer according to a standard protocol. The mean of these three blood pressure measurements was used in the data analysis. Height and body weight were measured by trained staff using a balance beam scale after subjects removed their shoes and were wearing light clothing. Body mass index (BMI) was calculated as weight in kilograms divided by the square of the height in meters (kg/m^2^). Waist circumference was measured at the level of 1 cm above the umbilicus.

Overnight fasting blood samples were obtained to measure cholesterol, blood glucose, insulin, high sensitivity C-reactive protein (hsCRP), soluble intercellular adhesion molecule-1 (sICAM-1), soluble E-selectin (sE-selectin) and angiotensin II. Plasma and serum samples were frozen at -80°C until laboratory testing. Concentrations of total cholesterol, high density lipoprotein (HDL) cholesterol, and triglycerides were assessed enzymatically on a Beckman Synchrony CX5 Delta Clinical System (Beckman Coulter, Fullerton, CA, USA) using commercial reagents, and low density lipoprotein (LDL) cholesterol concentration was calculated by means of the Friedewald equation for participants who had less than 400 mg/dL triglycerides [32]. Fasting blood was measured using a modified hexokinase enzymatic method. Concentration of hsCRP was determined by an immunoturbidimetric assay on a Beckman Synchron CX5 Delta Clinical System using commercial reagents. Soluble E-selectin and sICAM-1 was measured by an ELISA assay (R & D Systems, Minneapolis, MN) which employs the quantitative sandwich enzyme immunoassay technique. Quantitative determination of immunoreactive angiotensin-II was performed by a double antibody radioimmunoassay (RIA) after reversed-phase sample extraction by means of phenylsilylsilica columns following the method previously reported by Emanuel et al and a commercial RIA kit [33]. In total 178 people were missing a value for 1 of the 4 biomarkers. Values were imputed by using the median value of the biomarker.

### Definitions

Participants who smoked at least 1 cigarette daily for 1 year or more were defined as current smokers. Heavy alcohol consumption was defined as drinking at least 50 g of alcohol per day for 1 year or more. MetS was defined according to the modified Adult Treatment Panel III recommendations for Asian American [34]. Individuals were considered to have MetS if they had three or more of the following risk factors: elevated waist circumference (≥90 cm in men, ≥80 cm in women), elevated triglycerides (≥150 mg/dL), reduced HDL cholesterol (< 40 mg/dL in men, < 50 mg/dL in women), elevated BP (≥130 mmHg systolic blood pressure or ≥85 mmHg diastolic blood pressure) or elevated fasting glucose (≥100 mg/dL). Prediabetes and diabetes cases were defined as individuals with fasting blood glucose measurements of 100-125 mg/dL and ≥126 mg/dL, respectively.

### Statistical Analysis

Participants were categorized into one of four groups based on the presence or absence of metabolic and glycemic abnormalities: 1) free of prediabetes, diabetes and MetS (reference group), 2) prediabetes or diabetes only, 3) MetS without prediabetes or diabetes, and 4) MetS plus prediabetes or diabetes. Descriptive statistics for demographic variables, components of MetS, and biomarkers of inflammation and endothelial dysfunction were calculated separately for the four groups. For continuous variables, means (standard deviations [SD]) were used to describe normally distributed variables and median (interquartile range) were calculated for variables with skewed distribution; frequencies were used to describe categorical variables. Differences between the reference group and other groups were tested using ANOVA for normally distributed continuous variables; the Wilcoxon rank-sum test for variables that did not follow a normal distribution and χ^2 ^for categorical variables.

For each biomarker, participants were ranked according to concentration of the biomarker and then divided into tertiles. Mean concentrations of individual components of the MetS were calculated according to tertile of biomarker in age and gender-adjusted models and linear trends were tested. The distribution of participants was calculated by tertile of biomarker and category of metabolic or glycemic abnormality. Finally, multivariable logistic regression was used to calculate the odds ratio for prediabetes or diabetes, MetS without prediabetes or diabetes, or MetS plus prediabetes or diabetes according to tertile of plasma biomarker concentration. Consistent with other studies, we defined elevated biomarker concentration as the upper tertile of the distribution [17]. All p-values were 2-tailed and a significance level of 0.05 was used. All statistical analyses were conducted using SAS statistical software (version 9.2).

## Results

The descriptive characteristics of 2,536 study participants are presented separately for the four categories of metabolic or glycemic abnormalities (table 1). Study participants had a mean (SD) age of 46.4 (12.4) years, mean BMI of 22.2 (3.5) kg/m^2^, and 60% were female. In total, 3.6% of the study participants had diabetes, and 18.5% had prediabetes. Of those with prediabetes or diabetes, 61% also had the MetS and 27.4% of the entire study population had 3 or more components of the MetS. Compared to the reference group, participants with the MetS were more likely to be female, have elevated BMI, and have a family history of hypertension. In univariate analysis compared to the reference group, hsCRP was elevated in all groups, sICAM-1 was elevated among those with prediabetes or diabetes, and sE-selectin was elevated in those with MetS.

**Table 1:** Descriptive characteristics of 2,536 participants according to category of metabolic or glycemic abnormality

	No Prediabetes, Diabetes, or MetS	Prediabetes or Diabetes only	MetS without Prediabetes or Diabetes	MetS plus Prediabetes or Diabetes
No.	1621	218	354	343
Age, y	45.3 (12.4)	45.4 (12.8)	48.6 (10.7)*	50.4 (12.2)*
Female, %	57.2	50.1	67.0*	66.2*
Current smoker, %	46.8	42.2	39.3*	39.1*
Alcohol consumption, %	33.4	33.0	33.6	32.4
Body mass index, kg/m^2^	21.4 (2.9)	21.0 (3.2)	25.0 (3.3)*	24.3 (3.8)*
Family history of HTN, %	9.9	18.8*	14.4*	22.5*
**Components of the Metabolic Syndrome**				
HDL-C, mg/dL	47.6 (12.3)	48.5 (16.2)	38.3 (8.0)*	39.8 (12.2)*
Triglycerides, mg/dL	86.8 (61.2)	79.6 (40.0)	189.2 (180.7)*	172.7 (177.1)*
Waist circumference, cm	78.1 (7.9)	76.6 (6.8)*	89.2 (8.9)*	87.6 (10.2)*
Systolic BP, mmHg	124.7 (23.2)	126.1 (22.3)	141.5 (23.4)*	142.9 (25.1)*
Diastolic BP, mmHg	81.8 (12.2)	82.1 (11.8)	92.4 (11.3)*	90.5 (12.6)*
Glucose, mg/dL	81.5 (10.8)	112.5 (14.5)*	84.4 (10.2)*	119.7 (34.2)*
**Biomarkers of Inflammation and Endothelial Dysfunction**				
hsCRP, mg/L	5.13 (3.61, 8.25)	7.32 (3.64, 13.84)*	7.89 (5.19, 16.08)*	12.59(5.89, 20.4)*
sICAM-1, ng/mL	315.3 (93.4)	360.3 (85.2)	325.7 (94.7)*	377.6 (89.5)*
sE-selectin, ng/mL	17.9 (14.4, 23.6)	18.3(15.6, 22.3)	20.9 (16.1, 27.6)*	19.9 (16.6, 25.0)*
Angiotensin II, pg/mL	49.4 (39.5, 71.1)	46.7(40.0, 60.0)	50.5 (41.0, 73.8)	49.0(40.0, 86.0)

Abbreviations: BP, blood pressure; HDL-C, high density lipoprotein-cholesterol; hsCRP, high sensitivity C-reactive protein; sICAM-1, soluble intercellular adhesion molecule-1; sE-selectin, soluble E-selectin. Metabolic syndrome (MetS) was defined according to the revised ATP-III criteria defined as the presence of 3 or more of the following risk factors: waist circumference ≥90 cm for men and ≥80 cm for women, triglyceride concentration ≥150 mg/dL, blood pressure ≥130/85 mmHg, fasting glucose ≥ 100 mg/dL, or HDL cholesterol level < 40 mg/dL in men or < 50 mg/dL in women. For normally distributed variables, values are presented as means (SD) or frequencies (%) and as median (interquartile range) for variables that did not follow a normal distribution (hsCRP, sE-selectin, and angiotensin II). P-values were computed for each category versus the reference category using ANOVA or chi-square for normally distributed variables or Wilcoxon nonparametric rank test for variables that did not follow a normal distribution, values < 0.05 are denoted with an asterisk (*).

Table 2 displays the mean levels of individual components of the MetS presented by tertile of biomarker in a model adjusted for age and gender. HsCRP and sICAM-1 were associated with all components of the MetS (p-value for linear trend ≤0.01 for all components). Soluble E-selectin had a linear association with blood glucose, waist circumference, and triglycerides (p-value < 0.01 for linear trend). Angiotensin II was linearly associated with blood glucose, triglycerides, and blood pressure (p-value < 0.01 for linear trend).

**Table 2:** Mean levels (95% confidence intervals) of individual components of the metabolic syndrome by tertile of biomarker in a model adjusted for age and gender

Tertiles of Biomarker	Median	Blood Glucose (mg/dL)	Waist Circumference (cm)	Triglycerides (mg/dL)	HDL Cholesterol (mg/dL)	Systolic Blood Pressure (mmHg)	Diastolic Blood Pressure (mmHg)
**hsCRP (mg/L)**							
1	3.3	84.4 (83.0, 85.9)	78.0 (77.4, 78.7)	81.2 (73.8, 88.7)	47.7 (46.9, 48.6)	127.5 (126.0, 129.0)	83.2 (82.4, 84.0)
2	5.9	86.1 (84.7, 87.5)	80.9 (80.3, 81.5)	99.7 (92.4, 107.0)	45.7 (44.9, 46.6)	128.4 (126.9, 129.9)	84.2 (83.4, 85.0)
3	15.2	98.2 (96.8, 99.6)	83.7 (83.1, 84.3)	162.9 (155.7, 170.2)	42.4 (41.5, 43.2)	133.6 (132.2, 135.2)	87.1 (86.3, 87.9)
P-value for linear trend		< 0.001	< 0.001	< 0.001	< 0.001	< 0.001	< 0.001

**sICAM-1 (ng/ml)**							
1	234.3	84.2 (82.8, 85.7)	79.8 (79.2, 80.5)	110.9 (103.3, 118.6)	46.7 (45.8, 47.6)	127.7 (126.2, 129.2)	84.0 (83.1, 84.8)
2	329.0	87.8 (86.4, 89.3)	80.7 (80.6, 81.3)	109.9 (102.3, 117.6)	45.1 (44.3, 46.0)	129.1 (127.5, 130.5)	84.5 (83.7, 85.3)
3	421.4	96.9 (95.4, 98.3)	82.2 (81.5, 82.9)	125.1 (117.5, 132.7)	43.9 (43.0, 44.7)	133.0 (131.5, 134.5)	86.1 (85.3, 86.9)
P-value for linear trend		< 0.001	< 0.001	0.01	< 0.001	< 0.001	0.001

**sE-selectin (ng/mL)**							
1	13.6	86.2 (84.8, 87.7)	79.0 (78.4, 79.7)	104.8 (97.1, 112.5)	46.0 (45.1, 46.9)	128.7 (127.2, 130.2)	83.8 (82.9, 84.6)
2	18.3	92.0 (90.5, 93.4)	81.4 (80.8, 82.0)	111.2 (103.6, 118.9)	45.0 (44.2, 45.9)	132.2 (130.7, 133.7)	85.9 (85.1, 86.7)
3	27.9	90.8 (89.4, 92.3)	82.3 (81.7, 82.9)	129.6 (122.1, 137.2)	44.7 (43.8, 45.5)	128.8 (127.3, 130.3)	84.9 (84.1, 85.7)
P-value for linear trend		< 0.001	< 0.001	< 0.001	0.04	0.49	0.20

**Angiotensin II (pg/mL)**							
1	36.0	90.8 (89.3, 92.3)	80.8 (80.1, 81.4)	104.1 (96.4, 111.8)	46.1 (45.2, 46.9)	127.1 (125.6, 128.6)	83.4 (82.6, 84.2)
2	48.8	90.4 (89.0, 91.9)	81.0 (80.3, 81.6)	113.2 (105.5, 120.8)	43.8 (43.0, 44.7)	129.7 (128.2, 131.2)	84.8 (84.0, 85.7)
3	89.5	87.9 (86.4, 89.3)	81.1 (80.4, 81.7)	128.3 (120.8, 135.9)	45.8 (44.9, 46.6)	132.9 (131.4, 134.3)	86.3 (85.5, 87.1)
P-value for linear trend		0.003	0.56	< 0.001	0.49	< 0.001	< 0.001

Abbreviations: hsCRP, high sensitivity C-reactive protein; sICAM-1, soluble Intercellular adhesion molecule-1; sE-selectin, soluble E-selectin.

**Figure 1:**
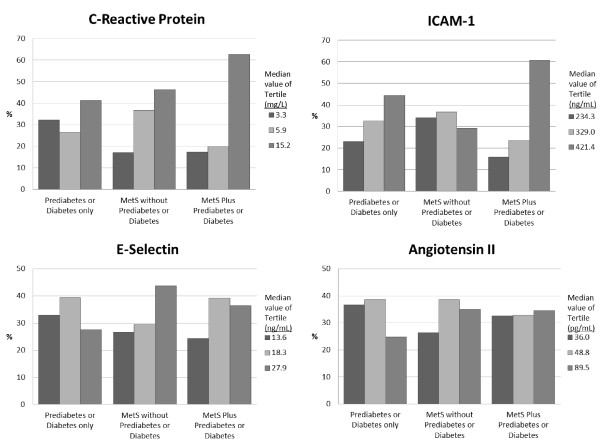
**Proportions of participants with prediabetes or diabetes only, metabolic syndrome without prediabetes or diabetes, and metabolic syndrome plus prediabetes or diabetes according to the tertile of biomarker concentration**.

Figure 1 shows the proportion of participants by tertile of biomarker and category of metabolic or glycemic abnormality. More than 40% of participants with prediabetes or diabetes only or MetS only have CRP in the upper tertile, whereas more than 60% of participants with MetS plus prediabetes or diabetes have elevated CRP. For ICAM-1, those in the top tertile comprised 44% of the individuals with prediabetes or diabetes only and more than 60% of those with MetS plus prediabetes or diabetes but only 29% of those with MetS only have elevated ICAM-1. For E-selectin, those in the top tertile comprised approximately 27% of those with prediabetes or diabetes only, 44% of those with MetS only, and 36% of those with the combination of the two conditions. The proportion of persons with elevated Angiotensin II was 25% in those with prediabetes or diabetes only, and 35% in the other two categories.

The age and gender-adjusted odds ratios ([OR], 95% confidence interval [CI]) for prediabetes or diabetes only, MetS without prediabetes or diabetes, and MetS plus prediabetes or diabetes are presented in table 3. Compared to the reference group, elevated hsCRP was associated with increased odds for all three groups; the increased risk was similar for those with prediabetes or diabetes only (2.27, 1.69-3.06), and MetS only (3.00, 2.35-3.84), but was significantly higher among those with MetS plus prediabetes or diabetes (5.84, 4.53-7.53). Elevated sICAM-1 was also associated with increased odds of prediabetes or diabetes (2.14, 1.60-2.86) and a 4-fold increased risk of MetS plus prediabetes or diabetes (4.15, 3.24-5.31). Elevated sE-selectin was associated with increased odds of MetS only (1.71, 1.35-2.17). Elevated Angiotensin II was not significantly associated with any of the outcome groups.

**Table 3:** Odds ratios for metabolic or glycemic abnormality associated with elevated biomarkers of inflammation and endothelial dysfunction

	No Prediabetes, Diabetes, or MetS	Prediabetes or Diabetes only	MetS without Prediabetes or Diabetes	MetS plus Prediabetes or Diabetes
**hsCRP (mg/L)**				
Age, gender	1.00	2.27 (1.69, 3.06)	3.00 (2.35, 3.84)	5.84 (4.53, 7.53)
Multivariable	1.00	2.22 (1.65, 3.00)	2.97 (2.33, 3.80)	5.70 (4.41, 7.33)
Multivariable + BMI	1.00	2.29 (1.69, 3.10)	2.38 (1.82, 3.11)	4.82 (3.69, 6.31)

**sICAM-1 (ng/ml)**				
Age, gender	1.00	2.14 (1.60, 2.86)	1.13 (0.88, 1.46)	4.15 (3.24, 5.31)
Multivariable	1.00	2.06 (1.54, 2.76)	1.10 (0.85, 1.43)	3.98 (3.10, 5.12)
Multivariable + BMI	1.00	2.11 (1.58, 2.83)	0.91 (0.69, 1.21)	3.43 (2.64, 4.47)

**sE-selectin (ng/mL)**				
Age, gender	1.00	0.82 (0.60, 1.12)	1.71 (1.35, 2.17)	1.27 (0.99, 1.62)
Multivariable	1.00	0.82 (0.60, 1.13)	1.71 (1.35, 2.17)	1.28 (1.00, 1.64)
Multivariable + BMI	1.00	0.85 (0.62, 1.17)	1.38 (1.06, 1.79)	1.06 (0.81, 1.38)

**Angiotensin II (pg/mL)**				
Age, gender	1.00	0.63 (0.45, 0.87)	1.09 (0.85, 1.39)	1.05 (0.82, 1.35)
Multivariable	1.00	0.63 (0.45, 0.87)	1.06 (0.83, 1.36)	1.00 (0.78, 1.29)
Multivariable + BMI	1.00	0.62 (0.45, 0.86)	1.12 (0.86, 1.46)	1.05 (0.80, 1.37)

Abbreviations: MetS, Metabolic Syndrome; hsCRP, high sensitivity C-reactive protein; sICAM-1, soluble Intercellular adhesion molecule-1; sE-selectin, soluble E-selectin; BG, blood glucose. The multivariable models are adjusted for age, gender, smoking, drinking and family history of hypertension.

When current smoking, heavy drinking, and family history of hypertension were added to the model, the results were modestly attenuated. Further addition of BMI to the model slightly attenuated most results but did not significantly change the overall results. Finally, there was no change in the overall results after excluding individuals with imputed data or excluding participants with CRP > 10 mg/L.

## Discussion

The primary findings of this study are that elevated levels of CRP and sICAM-1 are associated with increased odds of prediabetes or diabetes, MetS or both conditions in combination. Elevated CRP was associated with nearly 2-fold increased odds of prediabetes or diabetes, a 2-fold increased odds of MetS only, and nearly 5-fold increased odds of MetS plus prediabetes or diabetes after multivariable adjustment. Elevated sICAM-1 was associated with 2-fold increased odds of having prediabetes or diabetes, and nearly 4-fold increased odds of having MetS plus prediabetes or diabetes.

In the current study, 3.6% of the study participants had diabetes which is lower than recent national estimates but 18.5% had prediabetes which is slightly higher than national estimates [6]. Using the Revised ATP III definition of the MetS, approximately 27% of the present study participants had 3 or more metabolic abnormalities, which is similar to national estimates using the same definition[4].

Our findings are consistent with other studies that have reported a significant association between elevated CRP and prediabetes, diabetes [17,21,26] or MetS [21,28,30,35]. Our finding that sICAM-1 was associated with increased odds for prediabetes and diabetes is consistent with the association reported by the Women's Health Initiative Observational Study (OR for the highest verses lowest quartile of ICAM-1 values: 2.34, 95% CI 1.75, 3.13) but the odds were higher in the Nurse's Health Study (OR for the highest verses lowest quintile of ICAM-1 values: 3.56, 95% CI 2.28, 5.58), possibly due to analysis by quintiles rather than tertiles of biomarker concentration [18,19].

Levels of sE-selectin have been shown to be independently associated with diabetes and MetS [18-20]. Ingelsson et al. reported a 26% increased odds of MetS (OR 1.26, 95% CI 1.06-1.50) for each standard deviation increase in log E-selectin [20]. Unlike our study, the Women's Health Initiative Observational Study found E-selectin to be a predictor of diabetes; however, the U.S. women had higher median E-selectin levels, higher mean BMI and higher mean waist circumference than the women included in our study [19]. Angiotensin II has been thought to contribute to altering glucose and lipid metabolism by inhibiting formation of smaller, less insulin-resistant adipocytes. Although a positive linear association was identified between Angiotensin II and individual components of MetS, elevated angiotensin II levels were not associated with increased risk of MetS, prediabetes or diabetes only, or MetS plus prediabetes or diabetes. To our knowledge, no other studies have examined this association.

The mechanisms responsible for increased cardiovascular morbidity and mortality in individuals with diabetes or MetS, are not fully explained by traditional risk factors and may be partially explained by inflammation and endothelial dysfunction. Visceral adiposity is associated with increased production of proinflammatory cytokines and hepatic CRP. ICAM-1 is also associated with visceral adiposity and may be upregulated by the increased production of proinflammatory cytokines [36]. Inflammation may promote development of diabetes by triggering beta cell dysfunction, apoptosis and impaired insulin signaling or development of hypertension by influencing platelet adhesion and aggregation, production of oxidants and induction of renal vasoconstriction [37].

The median level of CRP in our study participants was higher than reported in other studies with Asian populations [26,28,30,35] and may be due to analytic or pre-analytic sources of variability such as a lack of standardized reference material for CRP measurement, inclusion of participants with CRP > 10 mg/L, diet high in fats and salts or subclinical or untreated disease.

This study has several strengths including homogeneity of lifestyle, dietary habits and ethnic background of study participants. Study personnel were well trained and data was collected with rigid quality control. Due to the cross sectional nature of this study, it is not possible to establish temporality or determine if elevated biomarkers lead to development of the prediabetes, diabetes or MetS or if development of the individual components of the MetS lead to inflammation and endothelial dysfunction. Additional limitation includes the fact that prediabetes and diabetes were defined based on the fasting glucose along, which will underestimate the prevalence [6].

## Conclusions

In conclusion, the results from our study, indicated a strong association between elevated biomarkers of inflammation and endothelial dysfunction with increased odds of prediabetes, diabetes and the MetS among adults in Inner Mongolia, China. Strategies to reduce inflammation may be important therapeutic targets for people at risk of developing diabetes or MetS.

## Competing interests

The authors declare that they have no competing interests.

## Authors' contributions

AMT analyzed the data and drafted the manuscript. YZ participated in the design and coordination of the study and helped to revise the manuscript. WT, TX, and LZ helped with acquisition of data and helped revised the manuscript. JC participated in the study design and coordination and helped to revise the manuscript. TNK helped with interpretation of data and revised the manuscript. CSC analyzed the data and revised the manuscript. LAB helped with the interpretation of data and to revised the manuscript. JH conceived of the study, participated in its design and coordination and helped to draft the manuscript. All authors read and approved the final manuscript.

## Pre-publication history

The pre-publication history for this paper can be accessed here:

http://www.biomedcentral.com/1472-6823/11/16/prepub
